# Celastrol inhibits store operated calcium entry and suppresses psoriasis

**DOI:** 10.3389/fphar.2023.1111798

**Published:** 2023-02-01

**Authors:** Xiaoman Yuan, Bin Tang, Yilan Chen, Lijuan Zhou, Jingwen Deng, Lin Han, Yonggong Zhai, Yandong Zhou, Donald L. Gill, Chuanjian Lu, Youjun Wang

**Affiliations:** ^1^ Beijing Key Laboratory of Gene Resource and Molecular Development, College of Life Sciences, Beijing Normal University, Beijing, China; ^2^ State Key Laboratory of Dampness Syndrome of Chinese Medicine, The Second Clinical College of Guangzhou University of Chinese Medicine, Guangzhou, China; ^3^ Guangdong-Hong Kong-Macau Joint Lab on Chinese Medicine and Immune Disease Research, Guangzhou University of Chinese Medicine, Guangzhou, China; ^4^ Department of Cellular and Molecular Physiology, The Pennsylvania State University College of Medicine, Hershey, PA, United States; ^5^ Key Laboratory of Cell Proliferation and Regulation Biology, Ministry of Education, College of Life Sciences, Beijing Normal University, Beijing, China

**Keywords:** psoriasis, celastrol, SOCE, STIM1, Orai1, CRAC channel, calcium

## Abstract

**Introduction:** Psoriasis is an inflammatory autoimmune skin disease that is hard to cure and prone to relapse. Currently available global immunosuppressive agents for psoriasis may cause severe side effects, thus it is crucial to identify new therapeutic reagents and druggable signaling pathways for psoriasis.

**Methods:** To check the effects of SOCE inhibitors on psoriasis, we used animal models, biochemical approaches, together with various imaging techniques, including calcium, confocal and FRET imaging.

**Results and discussion:** Store operated calcium (Ca^2+^) entry (SOCE), mediated by STIM1 and Orai1, is crucial for the function of keratinocytes and immune cells, the two major players in psoriasis. Here we showed that a natural compound celastrol is a novel SOCE inhibitor, and it ameliorated the skin lesion and reduced PASI scores in imiquimod-induced psoriasis-like mice. Celastrol dose- and time-dependently inhibited SOCE in HEK cells and HaCaT cells, a keratinocyte cell line. Mechanistically, celastrol inhibited SOCE *via* its actions both on STIM1 and Orai1. It inhibited Ca^2+^ entry through constitutively-active Orai1 mutants independent of STIM1. Rather than blocking the conformational switch and oligomerization of STIM1 during SOCE activation, celastrol diminished the transition from oligomerized STIM1 into aggregates, thus locking STIM1 in a partially active state. As a result, it abolished the functional coupling between STIM1 and Orai1, diminishing SOCE signals. Overall, our findings identified a new SOCE inhibitor celastrol that suppresses psoriasis, suggesting that SOCE pathway may serve as a new druggable target for treating psoriasis.

## Introduction

Psoriasis is a common autoimmune skin disease that affects about 2–3% of the world’s population (Rendon and Schäkel, 2019; Korman, 2020). Keratinocytes (Ortiz-Lopez et al., 2022) and immune cells are crucial players of psoriasis (Colombo et al., 2010; Steinckwich et al., 2015; Karczewski et al., 2016). Psoriasis is characterized by abnormal differentiation and hyper-proliferation of keratinocytes, as well as the release of innate immune-system activating factors from keratinocytes (Rendon and Schäkel, 2019). Activation of immune cells, particular Th1 and Th17 cells, and the functional imbalance of Th1 or Th17 over Tregs contribute to the progression of psoriasis (Heath et al., 2019). Similar to other types of autoimmune diseases, psoriasis is hard to cure and is prone to relapse. However, long-term usage of currently available medicines, like glucocorticoids and conventional immune-suppressants, often leads to serious side effects, including skin atrophy, hepatotoxicity, nephrotoxicity, and even cancer (Lee and Lee, 2018; Islam et al., 2019; Trebak and Kinet, 2019). For example, Methotrexate (MTX) is a WHO “essential medicine” widely employed as a first-line treatment in auto-immune, inflammatory diseases such as psoriasis. MTX probably alleviate psoriasis by inhibiting replication and function of T and B cells, suppressing the secretion of various cytokines such as interleukin 1 (IL-1), interferon-γ and tumor necrosis factor, and slowing down epidermal cell division (Yamauchi et al., 2003). However, psoriasis patients under long term MTX-therapy are at high risk of developing a liver injury. MTX-polyglutamic acid causes oxidative stress in the liver by inducing lipid peroxidation, which releases reactive oxygen species and inhibits antioxidant response elements. It also causes inflammation, steatosis, fibrosis and apoptosis (Ezhilarasan, 2021). Thus there is an urgent need for identifying new signaling pathways as targets for drug developments.

Calcium ion (Ca^2+^) is a ubiquitous second messenger, Ca^2+^ signals play fundamental roles in skin (Lee and Lee, 2018) and immune system (Trebak and Kinet, 2019). The store-operated Ca^2+^ entry (SOCE) mediated by Ca^2+^ release-activated Ca^2+^ (CRAC) channels is a major influx pathway in non-excitable cells (Parekh and Putney, 2005; Wang et al., 2008; Soboloff et al., 2012; Numaga-Tomita and Putney, 2013; Park et al., 2020). Classical CRAC channels are formed by Orai1 protein in the plasma membrane (PM) and stromal interaction molecules 1 (STIM1) in ER membrane (Hogan et al., 2010; Hogan and Rao, 2015). The lowering of the ER Ca^2+^ levels is first sensed by the N-terminal ER luminal domain of STIM1. Through allosteric conformational changes, the store-emptying information then is conveyed to its cytosolic coiled-coil 1 (CC1) region, which then unleash the STIM1-Orai1 activating region (SOAR) (Fahrner et al., 2014; Ma et al., 2015; Zhou et al., 2017; van Dorp et al., 2021; Shrestha et al., 2022). Activated STIM1 will accumulate at the ER-PM junctions, forming aggregates called puncta. Eventually, STIM1 will engage the pore-forming Orai1 protein *via* its SOAR region, constituting a protein complex or the CRAC channel, opening the Orai1-pore to allow Ca^2+^ influxes (Baraniak et al., 2020). CRAC channels are essential for the activation and proliferation of several types of T lymphocytes (Vaeth et al., 2020), and SOCE deficiency may cause severe combined immune deficiency disease in human (Prakriya and Lewis, 2015), genetic deletion of Orai1 or STIM1 attenuates cytokine production and Th17/Th1 cell-mediated diseases in model animals (Vaeth et al., 2020). CRAC channels are important players for the proliferation and differentiation of keratinocytes (Numaga-Tomita and Putney, 2013). Keratinocytes isolated from psoriasis patients showed a decreased SOCE response (Karvonen et al., 2000; Leuner et al., 2011). And recent findings have demonstrated that STIM1 depletion in neutrophils inhibits their capacity to infiltrate IMQ-induced psoriatic lesions in skin (Sethi et al., 2007). Therefore, targeting CRAC channels may be a promising approach to treat psoriasis and its recurrence.

Celastrol is a triterpene isolated from various species of the Celastracera including Tripterygium wilfordii Hook. f., the source of a traditional Chinese medicine (Cascao et al., 2017) (Liu et al., 2020). And it has shown its potential in treating various inflammatory and autoimmune diseases, such as inflammatory bowel disease (Jia et al., 2015; Zhao et al., 2015), skin inflammation (Kim et al., 2009a), systemic lupus erythematosus (SLE) (Li et al., 2005), rheumatoid arthritis (Goldbach-Mansky et al., 2009; Lv et al., 2015), osteoarthritis and allergy (Kim et al., 2009b; Ding et al., 2013; Cascão et al., 2017). However, there are still no reports regarding its effects against psoriasis. So far, some molecular targets of celastrol have been identified (Salminen et al., 2010; Chen et al., 2018a), and there are growing evidence showing its involvements of Ca^2+^ signaling. For example, it may increase cytosolic Ca^2+^ levels (Yoon et al., 2014; de Seabra Rodrigues Dias et al., 2018), possibly *via* activation of cannabinoid receptors (Jiang et al., 2020), or inhibition of sarcoplasmic/endoplasmic reticulum Ca^2+^ ATPase (SERCA) pump (Wong et al., 2019; Xu et al., 2020; Coghi et al., 2021). Overall, the full mechanistic spectrum underlying its actions still awaits further elucidation. Specifically, the possible effects of celastrol on SOCE, the aforementioned prominent Ca^2+^ entry process in immune cells and keratinocytes, still remain unexplored.

In the present study, we investigated the effects of celastrol on psoriasis and SOCE responses. We found that celastrol significantly ameliorated psoriatic skin lesion, reduced psoriasis area severity index (PASI) scores and improved skin immunopathology in imiquimod (IMQ)-induced psoriasis-like mice. This effect may be attributed to its inhibition on SOCE, probably *via* both preventing full-activation of STIM1 and some actions on Orai1. Our findings thus indicate that SOCE pathway may serve as a druggable target for treating psoriasis.

## Materials and methods

### Animals

BALB/c mice (male, 6–8 weeks old, 20 ± 2 g) were purchased from Guangdong Medical Laboratory Animal Center (Guangzhou, China). All mice were housed under standard laboratory conditions, fed with standard diet and provided free access to water. All animal experiments were approved by the Animal Experimental Ethics Committee of Guangdong Provincial Hospital of Chinese Medicine.

### Imiquimod (IMQ)-induced psoriasis-like mouse model and treatment

Forty BALB/c mice were randomly divided into five groups, including control, vehicle, celastrol low-dose (CEL-L), celastrol high-dose (CEL-H) and methotrexate (MTX). To establish a mouse model of psoriasis, an area of 3 × 2.5 cm of the back skin of the mice was first exposed, all mice except control group were then topically treated with 62.5 mg imiquimod (IMQ) cream on the back skin for seven consecutive days, as described previously (Chen et al., 2018b; Yue et al., 2019; Chen et al., 2020). The mice of celastrol-treated groups were orally administered with celastrol at a dose of 10 (CEL-L) or 20 (CEL-H) mg/kg/day for 10 consecutive days. For comparison, the mice of MTX group were orally administered with MTX at a dose of 1 mg/kg/day for 10 consecutive days. The mice of control and vehicle groups were only given the stroke-physiological saline solution daily for 10 consecutive days. The body weight and the psoriasis area severity index (PASI) scores of the mice were recorded on the first day of IMQ treatment for 7 consecutive days. Mice were sacrificed on day 10 and their blood, skin and spleen were collected for further analyses.

### Psoriasis area and severity index analysis and histological examination of skin

The severity of skin lesion was graded and monitored using an improved human scoring system, the psoriasis area severity index (PASI), which includes the area of the skin lesions, erythema, scaling and thickening, was measured and calculated for nine consecutive days. The PASI scores are 0 (none); 1 (light); 2 (moderate); 3 (severe); and 4 (extremely severe) (Pang et al., 2018). Skin samples from the mice were fixed in 4% neutral paraformaldehyde for 24 h and then embedded in paraffin. The samples in paraffin were cut into 7 μm-thick sections and stained with hematoxylin and eosin (H&E) for pathological observation with an optical microscope.

### Cell culture and transfection

Wild-type Human embryonic kidney 293 (HEK wt) cells or corresponding knockout cells: HEK SK (HEK STIM1-STIM2 double KO) and HEK OK (Orai1, Orai2 and Orai3 triple KO cells) (Wei et al., 2016), or HaCaT cells were routinely cultured in Dulbecco’s modified Eagle’s medium (HyClone, Chicago, IL, United States) supplemented with 10% FBS (cat: FBSSA500-S, AusGeneX, Australia) and 1% penicillin/streptomycin (Thermo Scientific, Waltham, MA, United States) (Zheng et al., 2018a). For HEK cells stably expressing Orai1-CFP or GCaMP6m, a highly sensitive Ca^2+^ indicator (Chen et al., 2013), regular DMEM medium supplemented with 100 μg/ml G418 (Invitrogen) were used. For HEK cells stably co-expressing Orai1-CFP and STIM1-YFP, both of 100 μg/ml G418 and 2 μg/ml puromycin (Invitrogen) were added to culture medium. All cells were kept at the presence of 5% CO_2_ at 37°C. The media were changed every 3–4 days and cells were passaged when confluent.

Gene transfection were performed as previously described (Li et al., 2020). Plasmids DNA were delivered to cells *via* electroporation using a voltage step pulse (4 mm cuvettes, 180 V, 25 ms, 0.4 ml OPTI-MEM), after which the cells were seeded on round coverslips and cultured in OPTI-MEM medium for another 30–60 min before DMEM medium was added. All experiments were carried out 24 h after transfection.

### Ca^2+^ imaging in living cells

All Ca^2+^ imaging assays performed in HEK cells were similar to those described before (Zheng et al., 2018a; Zhou et al., 2018; Dong et al., 2019; Yue et al., 2020). Briefly, intracellular Ca^2+^ imaging was conducted at room temperature using a ZEISS oberserver-Z1 microscope equipped with X-Cite 120-Q (Lumen Dynamics, Waltham, MA, United States) light source, ORCA-Flash4.0 V3 Digital CMOS camera (Hamamatsu, Japan), ×40oil objective (NA = 1.30), and standard Semrock filters, controlled with the SlideBook6.0 software (Intelligent Imaging Innovations, Inc.) using similar protocols as described previously (Wang et al., 2009). The Ca^2+^ free imaging bath solution contained 107 mM NaCl, 7.2 mM KCl, 1.2 mM MgCl_2_, 11.5 mM glucose, 20 mM Hepes-NaOH, pH 7.2. When needed, 1 μM thapsigargin (Sigma Aldrich) was added to the imaging solution to deplete ER Ca^2+^ store, 1 mM CaCl_2_ was added afterwards to induce Ca^2+^ influxes.

For intracellular Ca^2+^ responses shown by Fura-2 dye (cat#: F1201, Sigma Aldrich), the cells were first bathed in the imaging solution containing 2 μΜ Fura-2 AM for 1 h at room temperature in the dark to get Fura-2 AM loaded into cells and subsequently incubated in Fura-2 AM free imaging solution for another 30 min. The cytosolic Ca^2+^ signals were then acquired using a FURA2-C-000 filter set. Emission fluorescence signal at 510 ± 42 nm generated by light at the 340 ± 12.5 nm excitation wavelength (F_340_) and at 387 ± 5.5 nm (F_380_) was acquired every 2 s, the resulting F_340_/F_380_ ratio of Fura-2 were then converted to Ca^2+^ concentration *via* previously described calibration protocols (Ma et al., 2015; Dong et al., 2019) (Zheng et al., 2018a).

For intracellular cytosolic Ca^2+^ measured with a genetically encoded Ca^2+^ indicator (GECI), GCaMP6m (Chen et al., 2013), GCaMP6m fluorescence were obtained with GFP-1828A-000 filter set (457–484 nm _Ex_; 497–525 nm _Em_). The changes in intracellular Ca^2+^ levels are presented as changes in GCaMP6m fluorescence (ΔF/F_0_).

For measurements of ER Ca^2+^ levels with a ratiometric GECI named miGer (mKate covalently linked to a monomeric GECI named GCEPIA1er) (Li et al., 2020), G-CEPIA1er (457–484 nm _Ex_; 497–525 nm _Em_), and mKate fluorescence (539–557 nm _Ex_; 580–678 nm _Em_) were collected with Semrock filters (Cat#: FF01-470/22 _Ex_ or FF01-549/12 _Ex_, FF493/574 _Dic_, FF01-512/630 _Em_). And the ER Ca^2+^ levels are indicated by F_GCEPIA1er_/F_mKate_ ratio.

### Fluorescence or förster resonance energy transfer (FRET) imaging

For FRET measurements, the same system used in Ca^2+^ measurements was used, and necessary calibrations and offline analysis were performed as described before (Li et al., 2011). Experiments were performed in HEK293 cells stably expressing Orai1-CFP and STIM1-YFP, or transiently transfected with YFP-SOAR and STIM1_1-310_-CFP, or YFP-STIM1 and CFP-STIM1. The raw images (F_CFP_, F_YFP_, and F_raw_, respectively) were captured every 10 s at room temperature using the following three filters: CFP (438 ± 12 nm _Ex_/483 ± 16 nm _Em_), YFP (510 ± 5 nm _Ex_/542 ± 13.5 nm _Em_), and FRET _raw_ (438 ± 12 nm _Ex_/542 ± 13.5 nm _Em_). After raw images were obtained, the corresponding mean fluorescence from regions of interest were exported from the SlideBook6.0 software and imported into Matlab 2014a to calculate the system-independent apparent FRET efficiency, E_app_. The calculation methods used to generated E_app_ values from raw fluorescent signals were the same as those previously described (Ma et al., 2017) and the resulting data were plotted using the Prism 7 software. Representative traces from at least three independent experiments are shown as mean ± SEM.

### Confocal microscopy

Images were undertaken using a ZEISS LSM880 confocal system equipped with a ×63 oil objective (NA 1.4; Zeiss) and controlled by ZEN 2.1 software. CFP and YFP were excited by 405 and 514 nm laser respectively, and the resulting fluorescence was collected at 463–520 nm and 520–620 nm. The slice thickness is 1 μm. The acquired raw images were analyzed using ImageJ software (NIH). All experiments were repeated at least three times, and the representative data were shown.

### Cell proliferation essay

The proliferation speed of HaCaT cells were monitored and analyzed with IncuCyte Live Cell Analysis System (ESSEN BioSCIENCE), using standard protocols provided by the company. HaCaT cells were seeded into a 96-well dish with a density of 5,000 cells/well. Cells were grown in regular DMEM supplemented with either 0.1% DMSO (control) or 3 μM celastrol. After cells were fully attached, the 96-well dish was then loaded into IncuCyte and the images of cells in each well were recorded with proliferation protocol every 2 h. After 4–5 days, the proliferation rates of HaCaT cells was assessed by measuring the degree of cell fusion (Zhao et al., 2022).

### Real time polymerase chain reaction (RT-PCR) analysis

Total RNA was extracted from the samples with Trizol (Invitrogen, United States), and the RNA concentration was detected with an ultraviolet spectrophotometer (Beckman, DU-530, United States). According to the conditions provided in the instructions for the PrimeScript RT Reagent Kit (Takara, China), after denaturation at 95°C for 15 s, annealing at 61°C for 15 s, the RNA was reverse-transcribed into cDNA *via* a total of 35 cycles of amplification. Based on the SYBR Premix Ex Taq Ⅱ formulation (Takara, China), quantitative analysis was performed using a fluorescence quantitative polymerase chain reaction (PCR) instrument (Thermo Life, ViiA7, United States). The primer sequences used in this study are listed in Table 1. The relative mRNA quantities were determined by the 2^−ΔΔCT^ method (Lu et al., 2021), and further normalized to that of the GAPDH housekeeping gene.

**TABLE 1:** Sequences of primers.

Target	Forward	Reverse
TNF-α	ACT​GAT​GAG​AGG​GAG​GCC​AT	CCG​TGG​GTT​GGA​CAG​ATG​AA
IL-6	TTC​TTG​GGA​CTG​ATG​CTG​GT	CCT​CCG​ACT​TGT​GAA​GTG​GT
IL-17A	GTC​CAA​ACA​CTG​AGG​CCA​AG	ACG​TGG​AAC​GGT​TGA​GGT​AG
IL-23	AAT​AAT​GTG​CCC​CGT​ATC​CAG​T	GCT​CCC​CTT​TGA​AGA​TGT​CAG
P65	TGC​GAT​TCC​GCT​ATA​AAT​GCG	ACA​AGT​TCA​TGT​GGA​TGA​GGC
*β*-actin	GTG​ACG​TTG​ACA​TCC​GTA​AAG​A	GCC​GGA​CTC​ATC​GTA​CTC​C

### Western blotting

Skin tissue samples were extracted with Minute™ Total Protein Extraction Kit (Invent Biotechnologies, Eden Prairie, MN). Briefly, weigh 40 mg of skin tissue and cut it into small pieces (1 × 1 mm or smaller), transfer it to Filter Cartridges, add 80 mg of Protein Extraction Powder and 100 ul of Native Buffer, grind it with Plastic Rods for 3 min, then add 100 ul of Native Buffer again and grind it for 1 min. Afterwards, centrifuge (14,000 g) at 4°C for 1 min, and collect the supernatant in Collection Tubes to obtain the total protein samples. The total cellular protein concentration was quantified with a biscinchoninic acid kit. The proteins in each sample were resolved by 12.5% sodium dodecyl sulphate-polyacrylamide gel electrophoresis and transferred onto polyvinylidene difluoride membranes. The membranes were blocked with 3% bovine serum albumin at room temperature for 30 min. The blocked membranes were then incubated with various primary antibodies at 4°C overnight, followed by 1 h of incubation with various secondary antibodies (Lu et al., 2021). Finally, the proteins were detected with a Bio-Rad Imaging System (Bio-Rad Biosciences, Hercules, CA, United States).

## Results and discussion

### Celastrol exerted a protective effect against imiquimod (IMQ)-induced psoriasis

**FIGURE 1:**
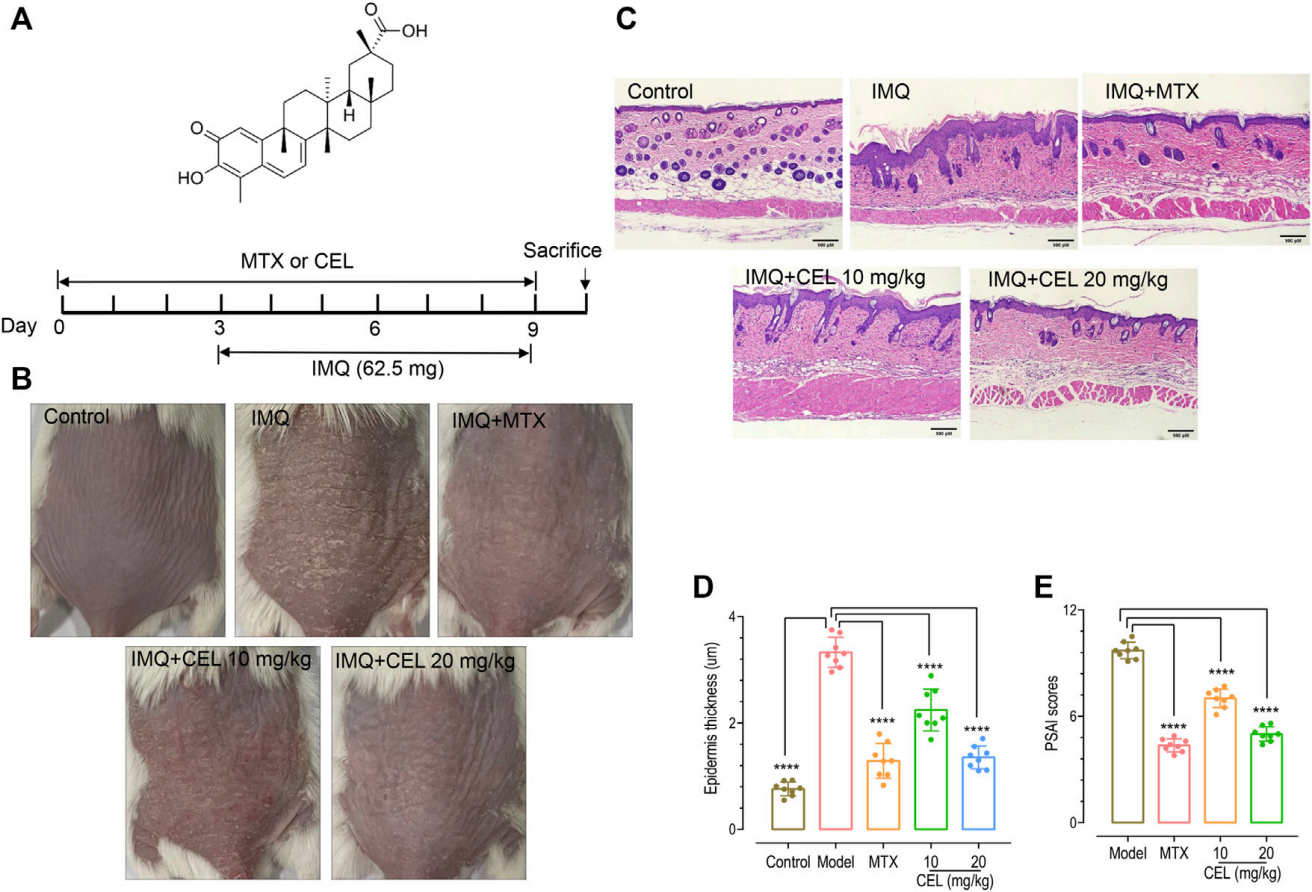
Celastrol ameliorated skin symptoms and inhibited epidermal hyperplasia in IMQ-induced psoriasis-like mice. **(A)** Structure of celastrol (top) and the timeline of various treatments (bottom). BALB/c mice were orally administered with celastrol (CEL-L: 10 mg/kg; CEL-H: 20 mg/kg) or MTX (1 mg/kg) for 10 consecutive days during the topical application of 65 mg IMQ on the dorsal skin. All mice were sacrificed on day 10. **(B)** Representative images of dorsal skin of mice on day 9. **(C)** Hematoxylin and eosin (H&E) staining of skin tissue from different groups of mice (magnification × 200, scale bar = 50 μm). **(D)** Epidermal thickness of mouse dorsal skin (****p < 0.0001 vs. IMQ-induced psoriasis group; *n* = 8). **(E)** Severity of psoriasis indicated by the psoriasis area and severity index (PASI).

To evaluate the effects of celastrol on psoriasis, we treated IMQ-induced psoriatic mice with celastrol. Methotrexate (MTX) was used as a positive control (Figure 1A). Similar to previous reports (Chen et al., 2018b), 7-day-IMQ treatments induced severe psoriasis-like skin lesions, including skin erythema, scales and thickness (Figure 1B, top two panels). Pre-treatments with celastrol (CEL-H, 20 mg/kg) dramatically attenuated the skin lesions of psoriatic mice (Figure 1B, bottom two panels), similar to those treated with MTX. We next asked whether celastrol could reduce epidermal hyperplasia, one typical pathological syndrome of psoriasis. We carried out hematoxylin and eosin (H&E) staining of mice back skin. Consistent with previous reports (Chen et al., 2018b), IMQ treatments led to a thickened epidermal spinous cell layer, parakeratosis and a large amount of inflammatory cell infiltration in the dermal layers. By contrast, IMQ-mice pretreated with MTX or celastrol showed reduced number of parakeratotic cells in the back skin lesions, a thinner epidermal spinous cell layer and a thinner epidermis (Figures 1C, D). Further analysis showed that celastrol- or MTX-treatments significantly decreased the scores of psoriasis area severity index (PASI) of IMQ-induced psoriatic mice (Figure 1E). Together, these results showed that celastrol can attenuate the psoriasis-like skin phenotype induced by IMQ in mice.

### Celastrol suppressed known upregulated inflammatory pathways that accompany IMQ-induced psoriasis

**FIGURE 2:**
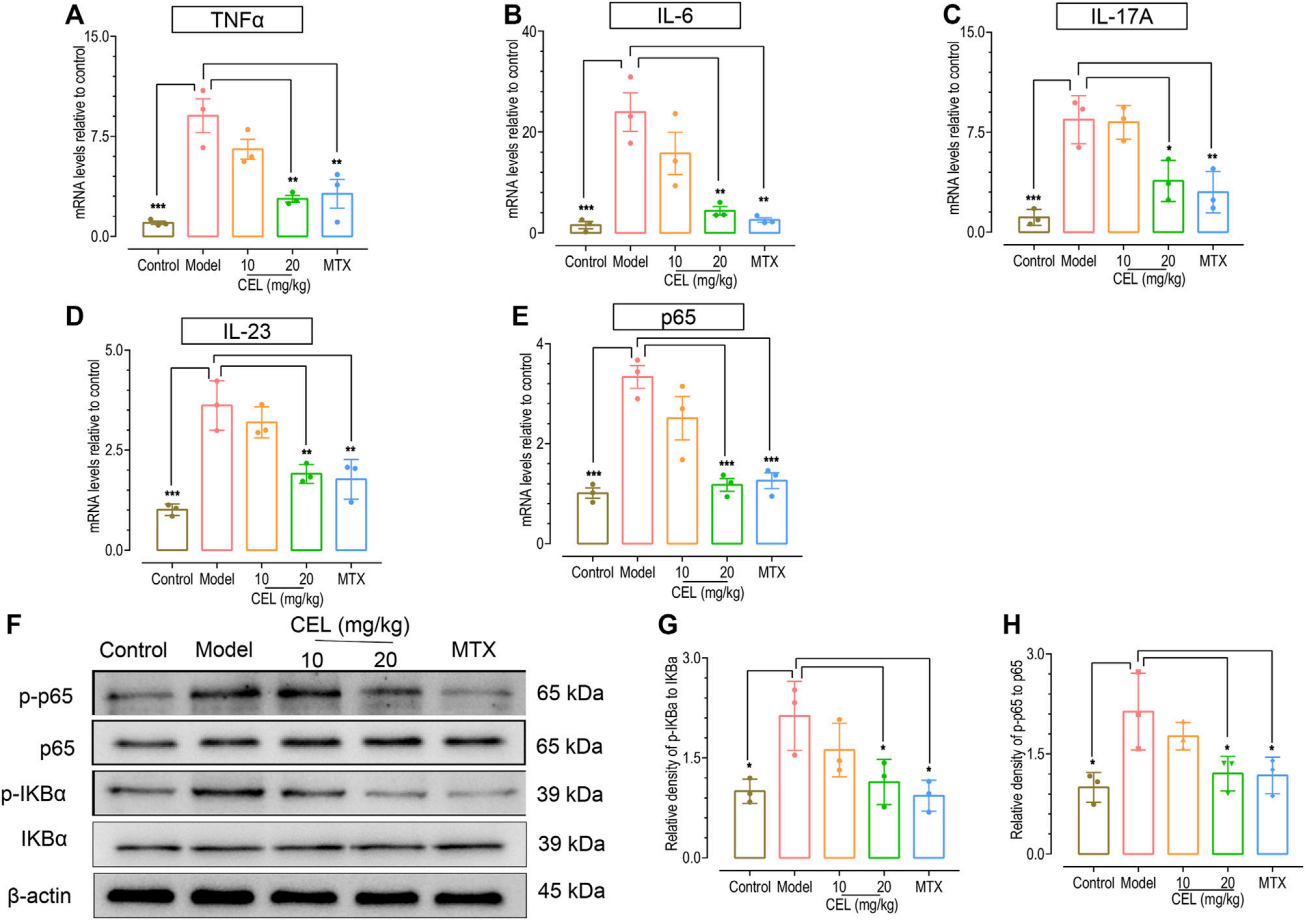
Celastrol attenuated the upregulation of proinflammatory cytokines and NFκB signaling in the skin of psoriatic mouse. The mRNA levels of TNF-α **(A)**, IL-6 **(B)**, IL-17 A **(C)**, IL-23 **(D)** and p65 **(E)** in the skin of IMQ-induced psoriasis-like mice determined by RT PCR analysis. **(F)** Representative WESTERN blotting images of p-IKBα, IKBα, p-NFκB p65 and NFκB p65. **(G,H)** Quantification of the relative expression of p-IKBα/IKBα and p-NFκB p65/NFκB p65, β-actin expression was used to normalize data. Values were expressed as fold changes relative to control group that was set as 1.0. Data of column graphs are presented as the mean ± SD from three separate experiments.

Psoriasis is accompanied by an increase in inflammatory cytokines such as interleukin IL-17A, IL-6, and Tumor Necrosis Factor *a* (TNFα) (Chen et al., 2018b), which are important triggers of inflammatory diseases (Onishi and Gaffen, 2010; Luo and Zheng, 2016; Yang et al., 2019). Thus we further measured the effects of celastrol on mRNA levels of these pro-inflammatory cytokines in the skin of IMQ-induced psoriatic mice using RT-PCR. Consistent with our previous observation (Chen et al., 2018b), the mRNA levels of IL-6, TNF-α and Th17 cytokines (IL-17A, IL-23) in the skin of psoriatic-like model mice were significantly higher than those in control group (Figures 2A–E). Similar to the effects of MTX, administration of celastrol, especially at high-doses, significantly diminished the increased mRNA levels of these pro-inflammatory cytokines of IMQ-treated mice.

We have previously shown that NFκB signaling is one upregulated crucial pathway involved in psoriasis-like skin inflammation (Chen et al., 2017). We thus examined the effect of celastrol on NFκB signaling, using MTX as a positive control. The results showed that both of them suppressed the phosphorylation of IKBα and P65 in the skin tissue (Figures 2F–H), indicating that both celastrol and MTX downregulate NFκB signaling in skin. Therefore, celastrol could inhibit the elevation of cytokine levels and the activation of NFκB pathway that accompanies IMQ-induced psoriasis.

### Celastrol inhibited SOCE in both HaCaT and HEK cells in a time- and dose-dependent manner

**FIGURE 3:**
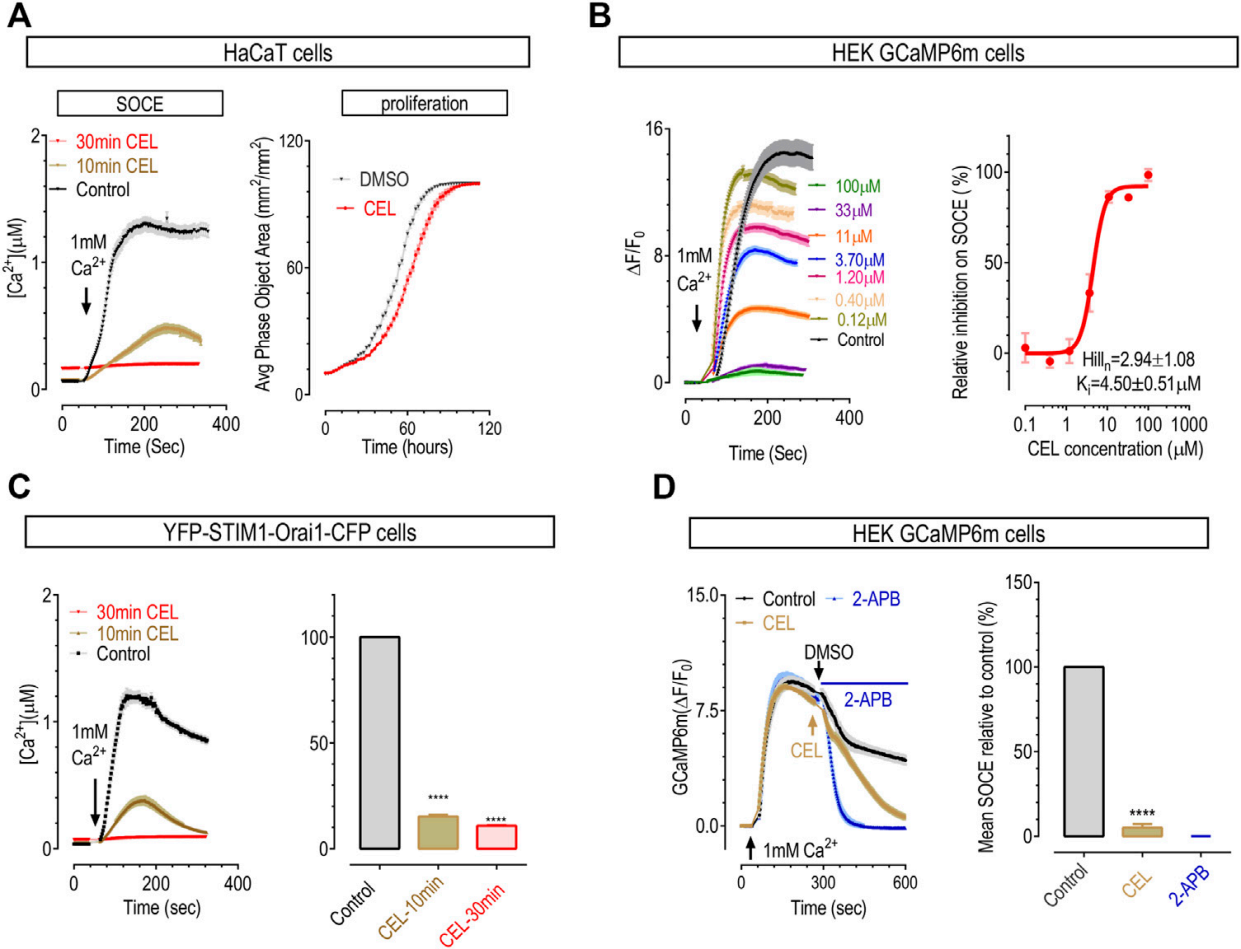
Celastrol dose- and time-dependently inhibited SOCE in HaCaT cells and HEK cells. To empty ER Ca^2+^ stores, prior to recordings, cells were first treated with 1 μΜ TG in Ca^2+^ free solution for 10 min. **(A)** The effect of pre-incubation of celastrol on SOCE and proliferation of HaCaT cells. Left, typical SOCE traces (50 μΜ); right, representative proliferation curves (3 μΜ). Doubling time: control, 18.9 ± 0.2 h; celastrol, 21.3 ± 0.3 h (*t*-test, ^****^, *p* < 0.0001, *n* = 3). **(B)** Effects of 10-min pre-incubation of celastrol on SOCE in HEK-GCaMP6m cells. Left, representative traces; right, dose response curves. **(C)** Action of celastrol on SOCE in HEK STIM1-Orai1 overexpressing cells. Prior to imaging, cells were pretreated 10 min (yellow), 30 min (red) with 50 μΜ celastrol or DMSO (black). Left, Typical traces; right, Statistics (*t*-test, ^****^, *p* < 0.0001, *n* = 3). **(D)** Effects of acute application of 50 μΜ celastrol on SOCE in HEK GCaMP6m cells. Effects of 50 μΜ 2-APB were used as a positive control. Left, Typical traces; right, Statistics (*t*-test, ^***^, *p* < 0.0001, *n* = 3).

Since celastrol affects many Ca^2+^ signaling processes and SOCE is the major Ca^2+^ influx route in non-excitable cells, we evaluated whether celastrol could also inhibit the SOCE responses of HaCaT cells, a human keratinocyte cell line with SOCE mediated by classical CRAC channels composed by STIM1 and Orai1 (Numaga-Tomita and Putney, 2013). A typical “Ca^2+^ add-back after store-depletion” protocol was used to induce SOCE (Zheng et al., 2018a). Briefly, the ER Ca^2+^ stores of HaCaT cells were first emptied by 10-min-bath in nominally Ca^2+^ free solution containing 1 μΜ thapsigargin (TG), a potent SERCA blocker. Afterwards, 1 mM Ca^2+^ were added back to external solution, and the resulting Ca^2+^ influxes through SOCE were monitored with Ca^2+^ imaging. As shown with Fura-2 Ca^2+^ indicator, HaCaT cells showed robust SOCE responses as previously described (Numaga-Tomita and Putney, 2013). 10 min incubation with 50 μΜ celastrol significantly inhibited SOCE responses, and longer treatments with celastrol totally abolish SOCE in HaCaT cells (Figure 3A, left panel). These results indicate that celastrol may also serve as an inhibitor for CRAC channels. Consistent with previous reports (Numaga-Tomita and Putney, 2013), inhibition of SOCE with celastrol significantly slowed down the proliferation of HaCaT cells (Figure 3A, right panel). Our observation, that celastrol suppressed the upregulated immune response pathways (Figure 2), also agreed with the notion that diminished SOCE would inhibit T-cell-mediated immune responses (Vaeth et al., 2020). Together, these results indicate that celastrol may alleviate psoriasis *via* inhibiting corresponding aberrant SOCE-dependent proliferation of keratinocytes and over-activation of immune cells.

To see whether this inhibitory effect is specific for CRAC response, or STIM1-Orai1 mediated SOCE, we examined the effect of celastrol on SOCE in HEK 293 cells stably expressing a genetically encoded Ca^2+^ indicator GCaMP6m (GCaMP6m cells). SOCE responses in HEK cells are mostly mediated by STIM1 and Orai1 (Zheng et al., 2018b). The results showed that, in GCaMP6m cells, 10 min incubation with 50 μΜ celastrol could dramatically inhibit SOCE (Figure 3B, left two panels), and the half-maximal inhibitory concentration (IC_50_) was 4.5 ± 0.5 μΜ (Figure 3B, the right panel). Thus celastrol could dose-dependently inhibit SOCE mediated by endogenous STIM1 and Orai1 in HEK cells. We then assessed the inhibitory effect of celastrol on SOCE mediated by exogenous STIM1 and Orai1 using fura-2-loaded HEK293 cells stably over-expressing STIM1 and Orai1 (STIM1-Orai1 cells). The result showed that (Figure 3C), 50 μM celastrol could also inhibit SOCE in a time dependent manner: 10-min incubation resulted in approximately 60% loss of SOCE, and almost complete loss of SOCE after 30-min incubation. Thus celastrol could also inhibit SOCE mediated by exogenous STIM1 and Orai1. Together these results showed that celastrol is an inhibitor for prototypical CRAC signals, or SOCE.

Even though celastrol did not significantly alter basal Ca^2+^ levels, we noticed that treatments with celastrol significantly increased the cytosolic Ca^2+^ levels in store-emptied cells, indicating an impairment of Ca^2+^ clearance. Indeed, the Ca^2+^ clearance rate was significantly inhibited in cells treated with 50 μΜ celastrol for 10 min (Supplementary Figure S1A), probably *via* its possible inhibition on Ca^2+^ pumps on PM. Nevertheless, it was reported that celastrol may inhibit SERCA (Wong et al., 2019; Xu et al., 2020; Coghi et al., 2021), probably resulting in ER Ca^2+^ store depletion. Indeed, when measured with miGer, a ratiometric ER Ca^2+^ indicator we recently developed (Li et al., 2020), celastrol could gradually decrease miGer ratio to a level that is similar to control cells treated with 2.5 μM ionomycin, an ionophore that can fully deplete ER store. Thus 50 μΜ celastrol could fully empty ER Ca^2+^ store of HEK cells (Supplementary Figure S1B). These results showed that celastrol may serve as a general blocker for calcium signaling at high concentration (50 μΜ).

We next performed more characterization on the inhibitory effects of celastrol on Ca^2+^ handling. At 10 μΜ, celastrol did not show effects on Ca^2+^ clearance or basal ER Ca^2+^ levels (Supplementary Figures S1C, D). While at this concentration, the inhibition on endogenous SOCE was already quite complete (Figure 3B), and 30 min incubation with 10 μΜ celastrol could significantly inhibit SOCE mediated by co-expressed STIM1 and Orai1 (Supplementary Figure S2A). Thus celastrol is a specific SOCE inhibitor at lower concentration (10 μΜ).

To gain more information about its time-dependent inhibition on CRAC signals, we checked the effect of celastrol-addition after SOCE reaching a plateau in GCaMP6m cells (Figure 3D). The blank control group showed some minimal inhibition on SOCE, likely caused by Ca^2+^ dependent inhibition, while 50 μM 2-APB could rapidly abolish SOCE as previously reported (Wei et al., 2016). As to the celastrol group, the results showed that it took about 5 min for celastrol to mostly diminish SOCE. This result, together with results showing its inhibition on SOCE reached maximal in around 30 min in HaCaT cells and STIM1-Orai1 cells (Figures 3A, C), suggest that celastrol’s inhibition on CRAC signals might not through direct binding and needs some time to develop.

To further dissect the molecular mechanism underlying celastrol’s inhibition on CRAC signals mediated by STIM1 and Orai1, we set out to examine the effects of celastrol on STIM1-Orai1 coupling, STIM1 and Orai1. To obtain more robust effects, we used the concentration of 50 μΜ, approximately 10-fold of K_i_ on SOCE.

### Celastrol disrupted the functional coupling between STIM1 and Orai1 by its actions on STIM1

**FIGURE 4:**
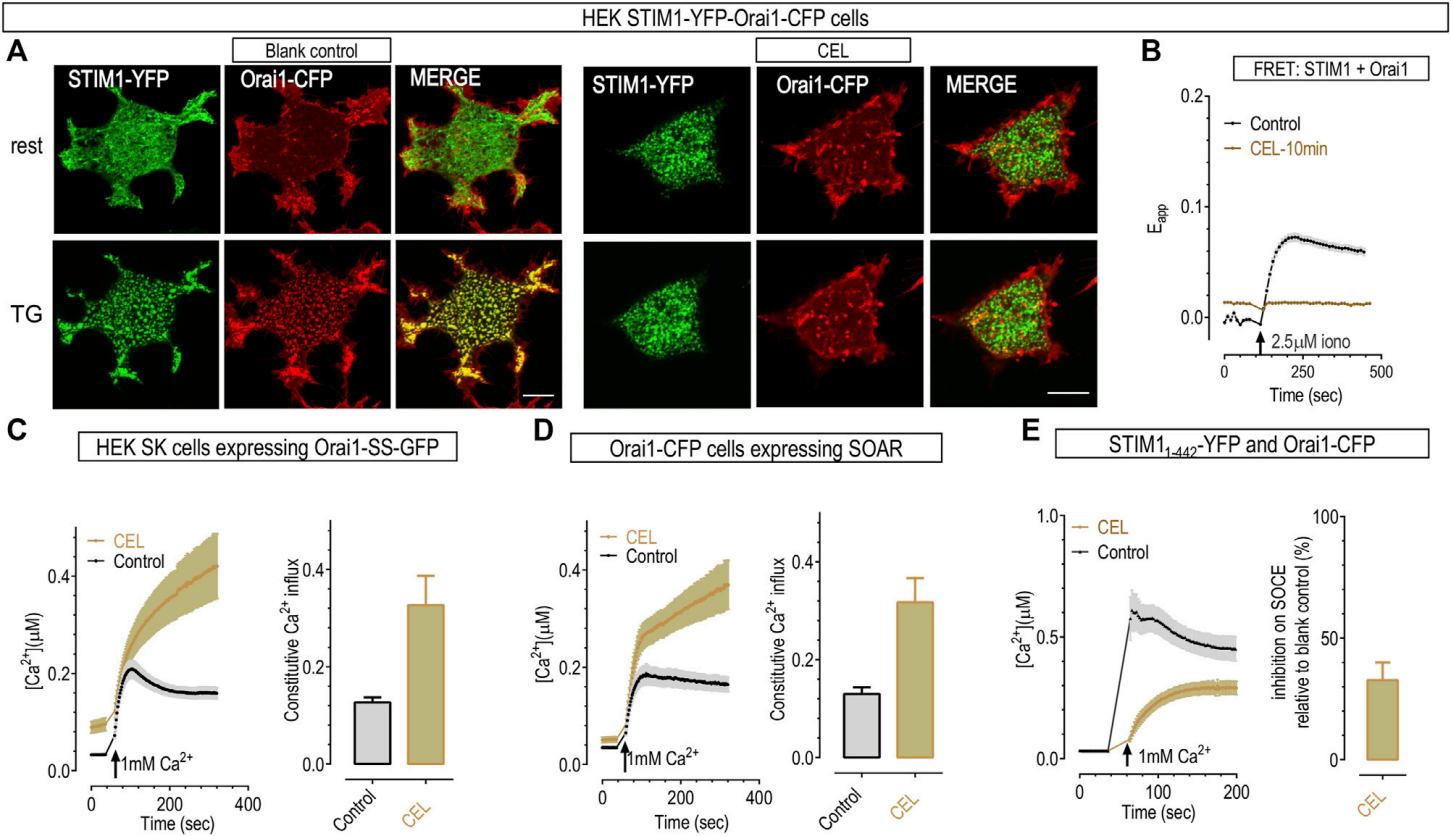
50 μΜ celastrol disrupted functional couplings between Orai1 and STIM1 but had no effect on Ca2+ influxes mediated by Orai1 and SOAR. **(A)** Typical confocal images showing the effect of 10-min celastrol on the colocalization between STIM1-YFP (green) and Orai1-CFP (red) in HEK STIM1-Orai1 cells. Top panels, store replete cells. Unlike control cells that show even distribution, 10 min-incubation with celastrol induced the formation of small STIM1 puncta at rest (upper images). Bottom panels, store-depleted cells after 10-min bath with 1 μΜ TG. STIM1 in control cells aggregated into large puncta, while STIM1 showed no-further aggregation in celastrol-treated cells. Scale bar, 10 μm. **(B)** Celastrol’s effects on FRET signals between STIM1-YFP and Orai1-CFP in HEK STIM1-Orai1 cells. Compared to control, pretreatment with celastrol resulted in a rise in basal FRET signal, and a decrease of the FRET signal after store depletion. Store-emptying ionomycin failed to induce any further increase in celastrol-treated cells. **(C,D)** Celastrol did not inhibit the constitutive Ca^2+^ entry mediated in HEK SK cells transiently expressing Orai1-SS, or co-expressing Orai1 and SOAR. Left, Typical traces; right, Statistics. **(E)** Pre-incubation with celastrol inhibited SOCE in cells co-expressing Orai1 and STIM1_1-442_. Prior to recordings, ER Ca^2+^ stores were emptied by bathing cells in Ca^2+^ free solution containing 1 μΜ TG for 10 min. Left, Typical traces; right, Statistics.

We first examined whether celastrol could disrupt STIM1-Orai1 coupling in STIM1-Orai1 cells with confocal imaging. Consistent with numerous previous reports, STIM1 molecules are evenly distributed across the ER and show no colocalization with Orai1 at rest. After ER Ca^2+^ store depletion with TG, STIM1 molecules form punctate structure and co-localize with Orai1 (Figure 4A, left panel) (Wei et al., 2016; Zhou et al., 2017). Interestingly, 10-min pretreatment with 50 μΜ celastrol induced small STIM1 puncta at rest. This probably is due to ER Ca^2+^ store depletion caused by its inhibition on SERCA (Wong et al., 2019; Xu et al., 2020; Coghi et al., 2021) (Supplementary Figure S1B). Nevertheless, after store depletion with celastrol and TG, STIM1 no longer co-localized with Orai1 in celastrol treated cells (Figure 4A, right panel), indicating impairments in the coupling between STIM1 and Orai1. We thus examined the FRET signals between STIM1-YFP and Orai1-CFP before and after store-depletion with ionomycin. Pre-incubation with celastrol resulted in a minimal rise in basal STIM1-Orai1 FRET signal (Figure 4B), the ionomycin-induced FRET increases were abolished by pre-incubation with celastrol, and the maximal FRET signal after store depletion is significantly lower than those of control cells (Figure 4B). Importantly, when tested with a concentration (10 μM) that was specific for SOCE responses (Supplementary Figures S1C, D, S2A), celastrol could still significantly inhibit the store-depletion-induced FRET-increases between STIM1 and Orai1. Together, these results showed that celastrol could inhibit the coupling between STIM1 and Orai1.

To examine whether this inhibition is on STIM1-Orai1 coupling *per se*, we checked the effects of celastrol on constitutive Ca^2+^ entry mediated by Orai1-SS in HEK STIM1-STIM2 double knockout cells (HEK SK cells). Orai1-SS is a construct with one Orai1 subunit fused with one dimer of STIM1_336-485_ cytosolic fragments named S. The SS dimer would bind with and fully activate Orai1 even when ER Ca^2+^ store is replete (Li et al., 2011). The results showed that the constitutive Ca^2+^ entry through Orai1 pre-coupled with SS fragments were not inhibited by 10 min incubation with 50 μΜ celastrol (Figure 4C). Similarly, celastrol also did not inhibit the constitutive Ca^2+^ entry mediated by Orai1 and the STIM1-Orai1 activating region (SOAR) (Figure 4D). SOAR is the minimal cytosolic STIM1 fragments (STIM1_344-442_) that can directly bind with and activate Orai1 regardless of ER Ca^2+^ levels (Yuan et al., 2009). Together, these results indicated that either Orai1-STIM1 coupling interface is not required for celastrol’s inhibition, or SOAR or S occupied the action sites for celastrol. Thus these results showed that celastrol did not diminish the engagement of STIM1 with Orai1 by directly interfering the STIM1-Orai1 coupling interface.

The engagement of SOAR, or STIM1_344-442_, with Orai1 is controlled by the STIM1_1-343_, the domain that is at the N-terminus of SOAR (Prakriya and Lewis, 2015). We thus checked whether adding STIM1_1-343_ back onto SOAR could restore the inhibition of celastrol on SOCE. The results showed that, pretreatment with 50 μΜ celastrol greatly inhibited TG-induced SOCE in cells co-expressing Orai1 and STIM1_1-442_ (Stathopulos et al., 2008) (Figure 4E). Thus celastrol may inhibit STIM1-Orai1 mediated Ca^2+^ influx *via* its actions on STIM1 domains that are at the N-terminal of SOAR.

### Celastrol blocked the transition from oligomerized STIM1 into puncta

**FIGURE 5:**
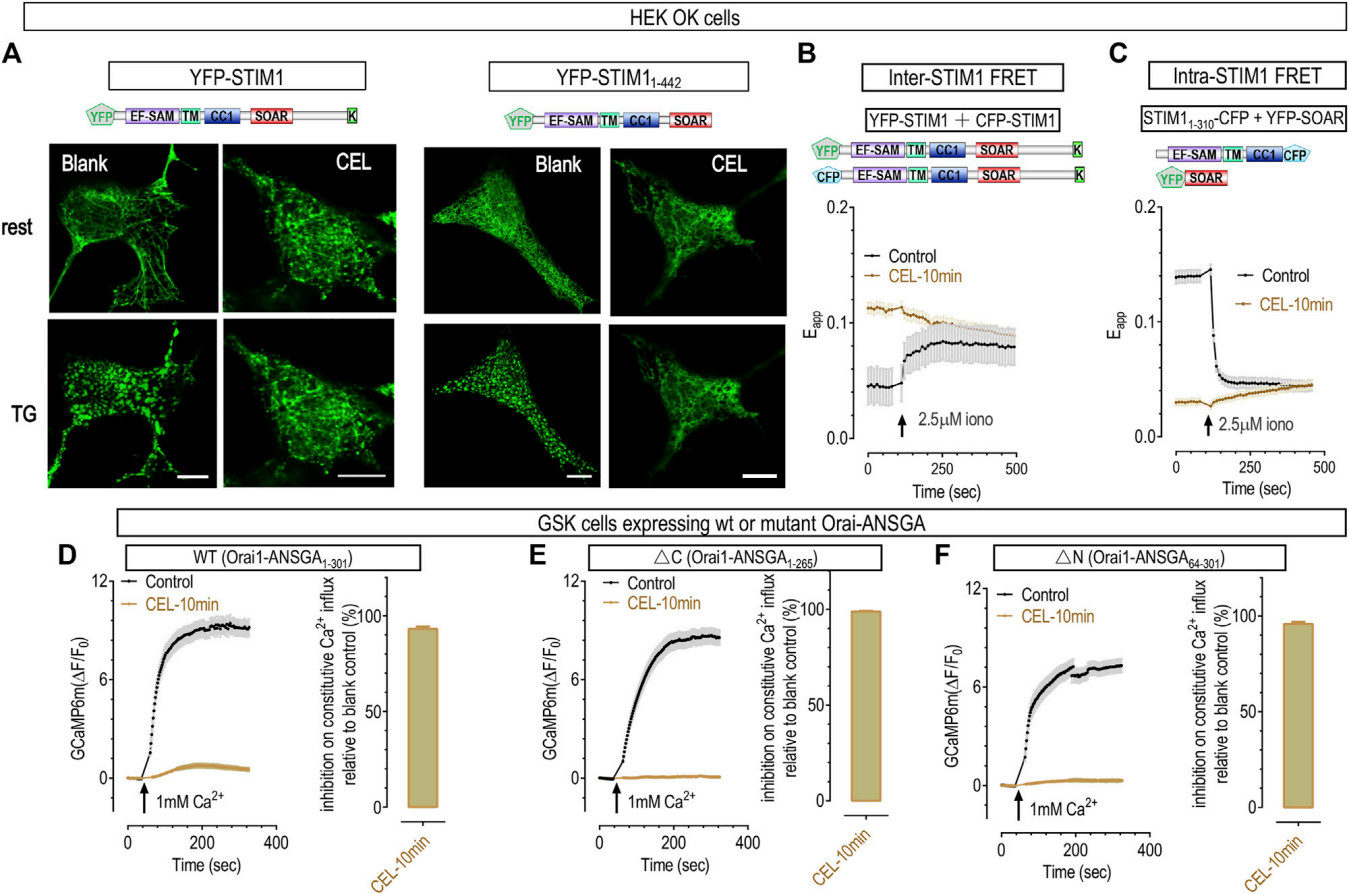
50 μΜ celastrol inhibited the formation of STIM1 puncta and Ca2+ influxes through constitutively active Orai1 mutants. **(A)** Representative confocal images showing the effects of celastrol on the store-dependent distribution of YFP-STIM1 or YFP-STIM1_1-442_ transiently expressed in HEK OK cells. Protocols used to induce store depletion by TG were the same as those in Figure 4A. For full-length STIM1 (Left two panels), unlike those in blank control cells (left most panels), celastrol-treatments induced minimal STIM1 punctate at rest and blocked the formation of store-dependent STIM1 puncta (right). As to YFP-STIM1_1-442_ (right two panels), they behave similarly to full length STIM1 in control cells (left), while celastrol completely blocked the formation of STIM1_1-442_ puncta (right). **(B)** Celastrol’s effects on FRET signals between YFP-STIM1 and CFP-STIM1 transiently co-expressed in HEK SK cells. Compared to control, pretreatment with celastrol resulted in a higher basal STIM1-STIM1 FRET signal, and abolished ionomycin-induced increases in this FRET signal. **(C)** Celastrol’s effects on FRET signals between STIM1_1-310_-CFP and YFP-SOAR transiently co-expressed in HEK SK cells. Compared to control, pretreatment with celastrol resulted in a significantly lower basal STIM1_1-310_- SOAR FRET signals, and abolished ionomycin-induced increases seen in control cells. **(D–F)** Celastrol’s effects on Ca^2+^ influxes mediated by constitutively active Orai1 mutants in GSK cells transiently expressing Orai1-ANSGA **(D)**, Orai1-ANSGA-ΔC _(1-265)_ **(E)** or Orai1-ANSGA-ΔN _(64-301)_ **(F)**. The constitutive Ca^2+^ influx were greatly inhibited by celastrol. Left, Typical traces; right, Statistics. At least three independent repeats were carried out for each experiments.

To further pinpoint the actions of celastrol on STIM1 in a clean back ground, we examined the behaviors of STIM1 in Orai1/2/3 triple knocked out (OK) HEK cells (Numaga-Tomita and Putney, 2013). Upon store depletion, STIM1 rearranges into puncta in ER-PM junctions, a signature of STIM1 activation (Figure 5A, left most panels) (Soboloff et al., 2012). In celastrol-treated OK cells, full-length STIM1 showed some punctate distribution, even after further treatments with TG to ensure full depletion of ER store (Figure 5A, second left panels). This is similar to what was observed in celastrol-treated STIM1-Orai1 cells, and consistent with the higher basal FRET signals between STIM1 and Orai1 (Figures 4A, B), all indicating a minimal activation of STIM1 after full depletion of the ER Ca^2+^ store (Supplementary Figure S1B). These results demonstrated that STIM1’s ability to form aggregated puncta after ER store depletion is greatly impaired.

It is intriguing that STIM1 could only form barely visible puncta in cells with empty ER Ca^2+^ stores. The C-terminus of STIM1 contains a poly-lysine (K) or polybasic (PB) domain that can bind with negatively charged lipids on PM and facilitate the formation of STIM1 puncta (Soboloff et al., 2012; Numaga-Tomita and Putney, 2013; Xu et al., 2020). We reasoned that celastrol may lock STIM1 at a partially active state: barely enough to engage its K region with PM to facilitate small puncta formation; but not enough to form large aggregates (Zheng et al., 2018b). We therefore examined the effect of celastrol on STIM1_1-442_, a truncated STIM1 construct that lack the K region in OK cells. Celastrol-treated OK cells expressing STIM1_1-442_ no longer showed puncta both at basal and TG-treated conditions (Figure 5A, right two panels). Together, these results clearly demonstrated that celastrol indeed diminish full activation of STIM1 after the emptying of ER Ca^2+^ stores.

During STIM1 activation induced by store depletion, it would first oligomerize and then aggregate more to form puncta. We thus asked whether the impaired ability of STIM1 to form puncta was caused by its inability to oligomerize after store-depletion with celastrol or TG. Firstly, we checked whether the ER luminal portion of STIM1 could still oligomerize in OK cells. The oligomerization status was indicated by FRET signals between STIM1 molecules with fluorescent proteins tagged at their N-terminus. Compared to those of control cells (Chen et al., 2018a), cells treated with celastrol showed higher basal inter-STIM1 FRET signals, and did not respond to further store-depletion with ionomycin (Figure 5B), consistent with the store emptying effect of celastrol. This result thus indicated that the luminal region of STIM1 oligomerized after store depletion with celastrol (Supplementary Figure S2B). Secondly, we examined whether the oligomerization of N-terminus STIM1 could propagate to its cytosolic side *via* its TM domain (Wei et al., 2016), by monitoring the FRET signals between STIM1_1-237_-CFP and STIM1_1-237_-YFP. In control cells, full store depletion with ionomycin could bring the TM region of STIM1 closer, resulting in an inter-STIM1_1-237_ FRET increase, initiating oligomerization of the cytosolic domain of STIM1 molecules (Wei et al., 2016). And celastrol could gradually increase the FRET signals to reach an amplitude that was similar to those in store-depleted control cells (Supplementary Figures S2C, D), indicating that the cytosolic side of STIM1 could respond to store depletion. Together, these results showed that celastrol did not alter the oligomerization ability of STIM1_1-237_ molecule during its activation. Therefore, STIM1 molecules could still sense the depletion of ER Ca^2+^ store in the presence of celastrol.

As celastrol had no effect on the oligomerization of activated STIM1_1-237_, or the ER-luminal and TM portion of STIM1, celastrol must inhibit the formation of STIM1_1-442_ puncta *via* its actions on STIM1_237-442_, or the CC1-SOAR domains of STIM1. The auto-inhibitory intra-molecular clamp between CC1-SOAR is known to keep STIM1 quiescent at rest, and the SOAR domain is essential for the formation of STIM1 puncta (Ma et al., 2015; Prakriya and Lewis, 2015; Wei et al., 2016). We thus utilized a FRET tool we developed for reporting the conformational switch during STIM1 activation (Ma et al., 2015), and examined whether celastrol had any effect on CC1-SOAR interaction. When co-expressed, ER-localized STIM1_1-310_ would bind the cytosolic SOAR and yield high basal FRET signals at rest. Upon store depletion with ionomycin, STIM1_1-310_ would unleash SOAR from ER to cytosol, resulting in diminished FRET signals (Karvonen et al., 2000; Shrestha et al., 2022). Similar to previous reports, ionomycin induced a significant decreases in FRET signals between STIM1_1-310_ and SOAR in control cells (Karvonen et al., 2000; Yue et al., 2019), indicating that STIM1 goes through a conformational transition from rest to activate configuration. Celastrol-treated cells showed significantly lower basal FRET signals that were similar to those in store-depleted control cells (Figure 5C), indicating that these two components of STIM1 already adopted an active configuration. This result is consistent with the store-emptying effect of celastrol (Supplementary Figure S1B), indicating that celastrol had no effect on the conformational switching of STIM1 during store depletion.

Overall, these results from both FRET measurements and confocal imaging showed that celastrol had no effect on conformational switching and oligomerization of STIM1 activation, but rather blocked the aggregation of activated STIM1 into puncta. Since SOAR domain is known to be essential for puncta formation and Orai1 activation (Ma et al., 2015; Prakriya and Lewis, 2015; Wei et al., 2016), it is likely that celastrol may inhibit the SOAR-mediated aggregation of STIM1. Further researches are needed to elucidate the possible mechanisms underlying this inhibition.

### Celastrol may also inhibit SOCE by its actions on Orai1

Lastly, we examined whether celastrol has any effect on Orai1 channels with Ca^2+^ imaging in HEK SK cells stably expressing GCaMP6m (GSK). To roll out possible interference by STIM1, we examined the effects of celastrol on Orai1-ANSGA, a constitutively active mutant that mediates authentic CRAC current independent of STIM1 (Zhou et al., 2016). Unlike constitutive Ca^2+^ entry mediated by Orai1-SS or SOAR-bound Orai1 (Figure 4E), the constitutive Ca^2+^ influxes through Orai1-ANSGA was greatly inhibited by 10 min-incubation with celastrol (Figure 5D). Similarly, celastrol nearly abolished the constitutive Ca^2+^ influxes mediated by its ΔC (Orai1-ANSGA_1-265_) version with no STIM1-binding ability (Zhou et al., 2016) (Figure 5E). Essential to keep Orai1-ANSGA constitutively active, the N-terminal Orai1_73-90_ is also known to weakly interact with STIM1 (Derler et al., 2013; Zhou et al., 2016). Therefore, it is likely that celastrol and STIM1 compete for interacting with Orai1_73-90_, and the binding of SS or SOAR with Orai1_73-90_ might block the inhibitory effect of celastrol (Figure 4E). Nevertheless, these results thus clearly demonstrate that celastrol also inhibits Orai1 channels independent of STIM1.

Orai1-mediated Ca^2+^ signaling pathways are fine-tuned, and the N-terminal region of Orai1 is crucial for its modulation. For example, PKC-mediated phosphorylation on Orai1-S27-S30 may inhibit SOCE (Zhou et al., 2018), and Orai1_17-37_ is crucial for SOCE to activate NFAT1 (Kar et al., 2021). We thus examined the effects of celastrol on N-terminus-truncated ANSGA (Orai1-ANSGA_64-301_), and found that celastrol could similarly diminish the constitutive Ca^2+^ entry in cells expressing Orai1-ANSGA_64-301_ (Figure 5F). Thus the action of celastrol on Orai1 is independent of its N-terminus region (Orai1_1-63_).

In summary, aiming to identify new reagents or signaling pathways for treating psoriasis, a hard-to-cure chronic inflammatory skin disease, here we showed that a natural compound celastrol has a therapeutic effect on psoriasis in a mice model. Mechanistically, celastrol is a novel inhibitor of CRAC channels. It inhibits SOCE by locking STIM1 at a partially-active state, and by its actions on Orai1_64-265_. Together, our findings suggest that SOCE may serve as a target for the treatments of psoriasis, laying the groundwork for future treatment strategies of autoimmune and inflammatory diseases.

## Data Availability

The original contributions presented in the study are included in the article/Supplementary Material, further inquiries can be directed to the corresponding authors.

## Supplementary material

SUPPLEMENTARY FIGURE S1 Effects of high (50 μM) or low (10 μM) low concentration of celastrol on Ca2^+^ handling in HEK cells. **(A)** 50 μM celastrol decreased the speed of Ca2^+^ clearance in HEK GCaMP6m cells. Left, typical traces; right, statistics. Before recordings, cells were bathed in Ca2^+^ imaging solution containing 1 μM TG, 1 mM CaCl2, and 1mM GdCl3 for 10 min. 1 mM GdCl3 was used to block Ca2^+^ transfer across PM and lock TG- or celastrol-induced Ca2^+^ releases in the cytosol. After recording of the baseline, CaCl2 and GdCl3 were then replaced with 300 μM EGTA to allow Ca2^+^ clearance *via* Ca2^+^ pumps and Na+/Ca2^+^ exchangers on PM. **(B)** 50 μM celastrol emptied ER Ca2^+^ store in HEK cells stably expressing miGer, a ratiometric ER Ca2^+^ indicator (HEK miGer cells). Left, typical traces; right, statistics. **(C)** 10 μM celastrol had no effect on Ca2^+^ clearance in HEK GCaMP6m cells. Same protocols as those in **(A)** were used. **(D)** 10 μM celastrol had no effect on ER Ca2^+^ levels in HEK miGer cells. Left, typical traces; right, statistics.

SUPPLEMENTARY FIGURE S2 Effects celastrol on SOCE pathway in HEK cells. **(A)** 10 μM celastrol decreased SOCE responses in STIM1-Orai1 co-expressing HEK STIM1-Orai1 cells as indicated by Fura-2 indicator. Left, typical traces; right, statistics. Before recordings, cells were bathed in Ca2^+^ imaging solution containing 1 μM TG, 0 mM CaCl2, for 10 min. **(B)** Effects of 10 μM celastrol on FRET signals between STIM1-YFP and Orai1-CFP in HEK STIM1-Orai1 cells. Left, typical traces; right, statistics. **(C,D)** 50 μM celastrol increased the FRET signals between STIM11-237-CFP and STIM11-237-YFP to a level similar to those in store-depleted control cells. **(C)** Left, representative traces, right, statistics. **(D)** Statistics showing FRET signals at basal or store-depleted conditions. For all experiments performed in this figure, *N* = 3, ****, *p* < 0.0001, Student T-test.
