# Supplementary material for: Celastrol inhibits store operated calcium entry and suppresses psoriasis
Source: Front Pharmacol. 2023 Feb 1;14:1111798. doi: 10.3389/fphar.2023.1111798 (PMC9928759; doi:10.3389/fphar.2023.1111798)
Supplement: Supplementary file 1 [file Presentation1.PPTX]

## Slide 1
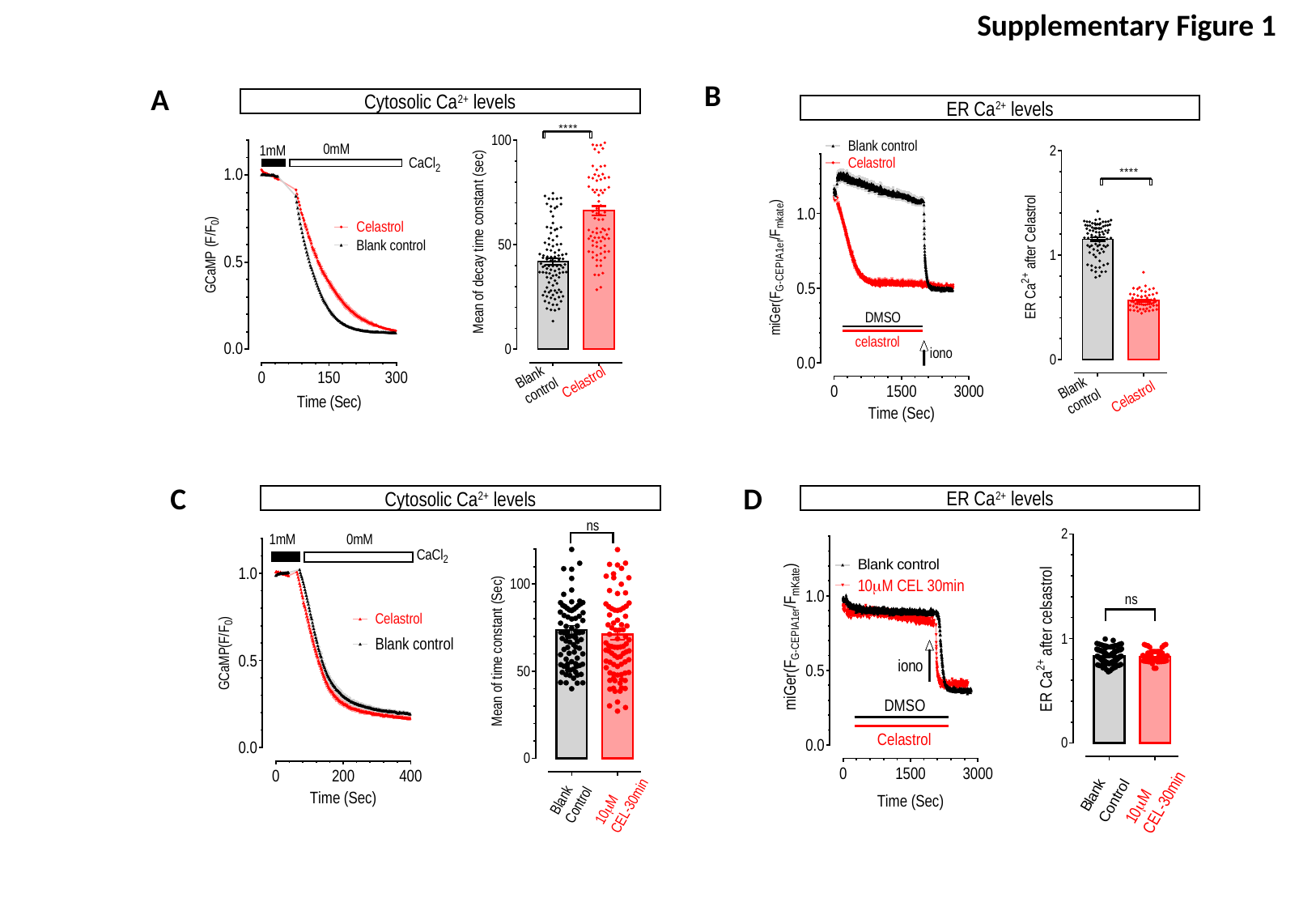

Supplementary Figure 1
B
A
Cytosolic Ca2+ levels
ER Ca2+ levels
C
D
ER Ca2+ levels
Cytosolic Ca2+ levels

## Slide 2
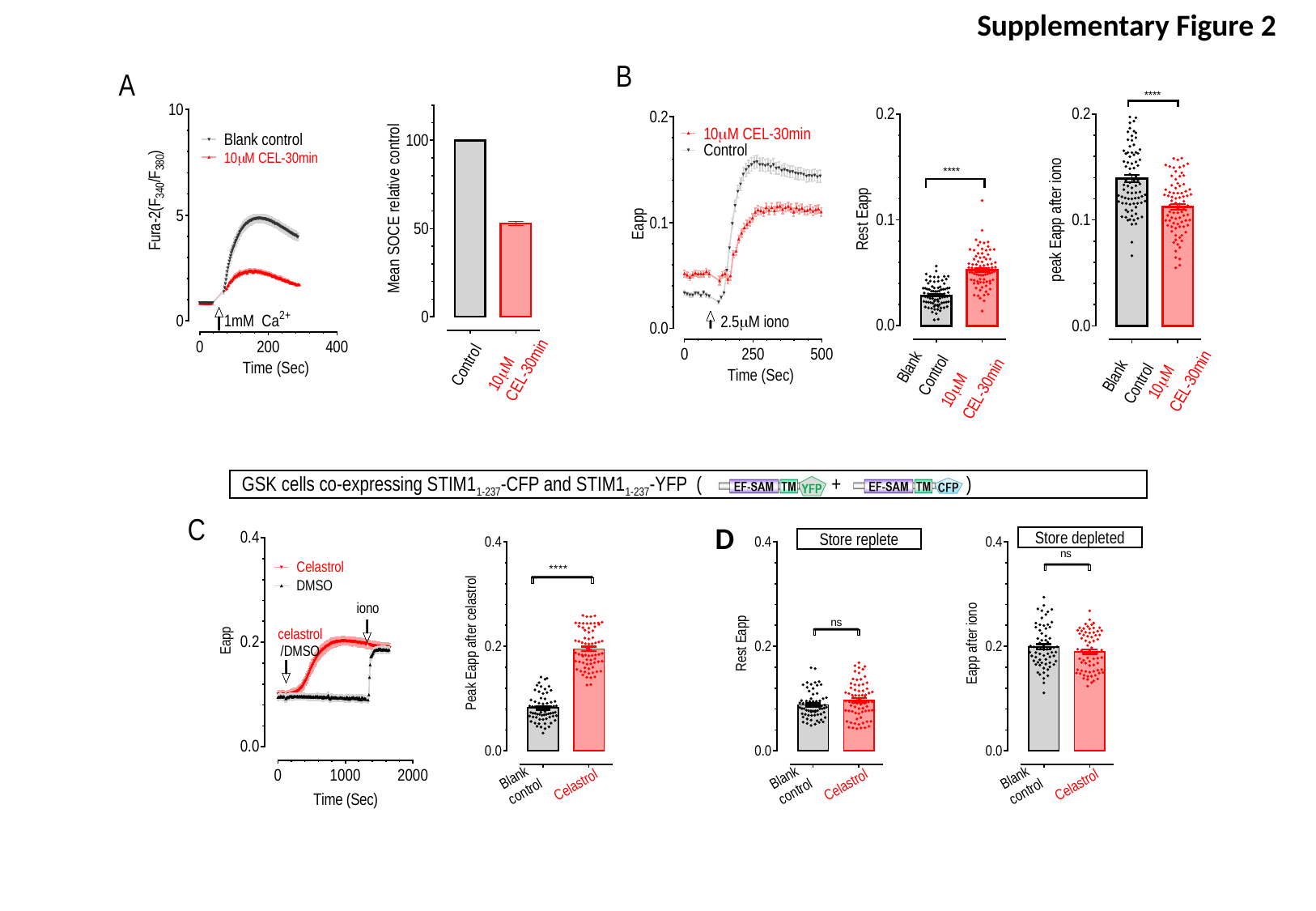

Supplementary Figure 2
B
A
GSK cells co-expressing STIM11-237-CFP and STIM11-237-YFP ( + )
C
D
Store depleted
Store replete
